# Serum Dysregulation of IL-36, IL-37, and IL-38 in Pyoderma Gangrenosum: Clinical Correlations and Implications for IL-36R-Targeted Therapy

**DOI:** 10.3390/ijms262412076

**Published:** 2025-12-15

**Authors:** Magdalena Łyko, Joanna Maj, Klaudia Rubas, Anna Ryguła-Kowalska, Danuta Nowicka-Suszko, Alina Jankowska-Konsur

**Affiliations:** 1 University Centre of General Dermatology and Oncodermatology, Wroclaw Medical University, 50-556 Wroclaw, Polandalina.jankowska-konsur@umw.edu.pl (A.J.-K.); 2 Student Research Group of Experimental Dermatology, University Centre of General Dermatology and Oncodermatology, Wroclaw Medical University, 50-556 Wroclaw, Poland; 3 Clinical Department of Otolaryngology, University Hospital, 50-556 Wroclaw, Poland

**Keywords:** pyoderma gangrenosum, IL-36, IL-37, IL-38, cytokines, inflammation, spesolimab, neutrophilic dermatoses

## Abstract

Pyoderma gangrenosum (PG) is a rare neutrophilic dermatosis characterized by chronic, painful ulcerations. Despite increasing evidence suggesting immunological dysregulation, the role of IL-36 cytokines in PG remains poorly defined. To evaluate serum levels of IL-36α, IL-36β, IL-36γ, IL-36Ra, IL-37, and IL-38 in PG patients compared to healthy controls, and to assess their correlation with selected clinical parameters and cytokine ratios. 44 PG patients and 40 healthy controls were included in this case–control study. Serum cytokine levels were measured using ELISA. Correlations between cytokine levels and clinical features were analyzed using nonparametric tests. PG patients showed significantly lower serum levels of IL-36α and IL-36γ (*p* = 0.0003 and *p* = 0.02, respectively), with no difference in IL-36β. Conversely, levels of IL-36Ra, IL-37, and IL-38 were significantly higher in PG patients (*p* < 0.0001 for all). In the PG group, significant positive correlations were observed between IL-36α and IL-36β, and between IL-36β and IL-36γ, while IL-37 correlated negatively with IL-38. IL-36α was inversely associated with serum IgA levels and total ulcer surface area, and IL-36γ correlated negatively with white blood cell count. Our findings reveal a dysregulated IL-36 cytokine profile in pyoderma gangrenosum, marked by reduced serum levels of IL-36α and IL-36γ and elevated levels of IL-36Ra, IL-37, and IL-38. This may reflect a compensatory response to chronic inflammation. The inverse correlation between IL-36α and ulcer size suggests its potential involvement in wound healing. Despite lower serum levels of agonists, local biological activity of IL-36 cytokines may remain elevated due to tissue-level activation and consumption. These results highlight the therapeutic relevance of targeting the IL-36 pathway—particularly in treatment-resistant cases—and support further research into cytokine activity beyond serum concentration to guide novel therapeutic strategies.

## 1. Introduction

Pyoderma gangrenosum (PG) is a rare neutrophilic dermatosis with estimated incidence of 3–10 cases per one million population per year. It is characterized by a sterile and progressively enlarging ulcer with an undermined border surrounded by erythema. It is also accompanied by pain [1,2]. One of the characteristic features is the pathergy phenomenon, which can be described as the rapid development of skin lesion frequently caused even by minor mechanical trauma [3,4]. The pathophysiology is poorly understood; nevertheless, altered neutrophil chemotaxis and loss of immune regulation, as well as autoinflammation or genetic factors, are considered [5]. PG is often associated with inflammatory bowel diseases (IBD), rheumatologic conditions, and hematologic disorders [6]. PG is also a part of some autoinflammatory syndromes such as PG, acne, pyogenic arthritis, and hidradenitis suppurativa (PAPASH); PG, acne, and pyogenic arthritis (PAPA); PG, acne, and hidradenitis suppurativa (PASH); psoriatic arthritis, PG, acne, and hidradenitis suppurativa (PsAPASH); PG, acne, and spondyloarthritis (PASS); and PG, acne, and ulcerative colitis (PAC) [7,8,9,10,11,12]

Interleukin (IL)-36 cytokines belong to the IL-1 superfamily and comprise three agonists (IL-36α, IL-36β, and IL-36γ) and three antagonists: IL-36 receptor antagonist (IL-36Ra), IL-37, and IL-38. These cytokines are key mediators in both innate and adaptive immunity and contribute to the pathogenesis of various autoinflammatory, autoimmune, and infectious diseases. In the skin, IL-36α and IL-36γ are predominantly produced by keratinocytes in the epidermis, but dendritic cells, macrophages, endothelial cells, and dermal fibroblasts also express them [13,14]. IL-36Ra functions as a competitive receptor antagonist that inhibits IL-36 signaling by binding to IL-1 receptor-like 2 (IL-1RL2/IL-36R) without inducing downstream effects. IL-37 and IL-38 are anti-inflammatory cytokines that act via broader mechanisms to limit excessive immune activation and maintain homeostasis [15]. IL-36 level can be dysregulated in inflammatory diseases such as generalized pustular psoriasis, rheumatoid arthritis or IBD [16]. However, in contrast to the abovementioned diseases, a comprehensive profile of IL-36 agonists and antagonists in PG, particularly in serum, remains unexplored.

The complex regulation of IL-36 cytokines through proteolytic activation and the opposing actions of endogenous antagonists such as IL-36Ra, IL-38, and IL-37 highlight the delicate balance of pro- and anti-inflammatory signals in neutrophilic dermatoses. Thus, elucidating this balance in PG not only offers new insights into its immunopathogenesis but is also crucial for validating the targeting of the IL-36 pathway as a rational therapeutic strategy. To investigate this, we evaluated the expression of IL-36α, IL-36β, IL-36γ, IL-36Ra, IL-37, and IL-38 in the serum of PG patients and correlations between the studied interleukins and clinical characteristics.

## 2. Results

The study group consisted of 29 women (65.9%) and 15 men (34.1%) with PG, while the control group included 21 women (52.5%) and 19 men (47.5%). The mean age of patients with PG was 50.2 ± 16.4 years, whereas the mean age of the control group was 48.2 ± 13.0 years.

A total of 44 patients with PG and 40 HC were included in the study. The mean age of PG patients was 50.2 ± 16.4 years, and 65.9% were women. In the control group, the mean age was 48.2 ± 13.0 years, with 52.5% women. Basic and clinical characteristics of PG patients and HC are presented in Table 1.

### 2.1. Group Comparisons

**Figure 1:**
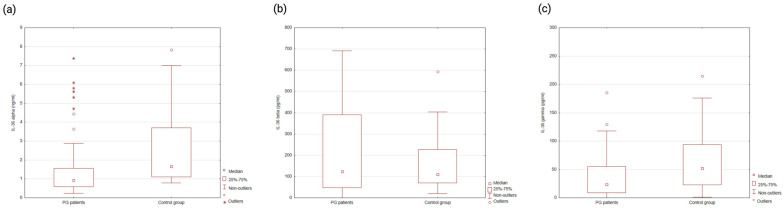
Serum levels of IL−36α (**a**), IL−36β (**b**), and IL−36γ (**c**) in PG patients and healthy controls. Statistically significant differences were observed for IL−36α and IL−36γ (*p* < 0.05), but not for IL−36β.

Using the Mann–Whitney U test, significantly lower serum levels of IL-36α (median: 0.93 ng/mL vs. 1.66 ng/mL; *p* = 0.0003) and IL-36γ (median: 23.76 pg/mL vs. 52.04 pg/mL; *p* = 0.02) were observed in PG patients compared to controls. No statistically significant difference was found in IL-36β levels (*p* = 0.58) (Figure 1).

**Figure 2:**
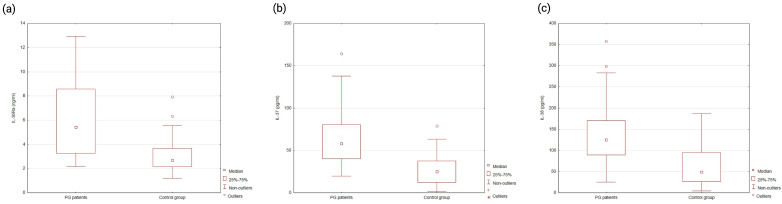
Serum levels of IL-36Ra (**a**), IL-37 (**b**), and IL-38 (**c**) in PG patients and healthy controls. All three cytokines were significantly elevated in PG patients compared to controls (*p* < 0.001 for each).

Conversely, serum concentrations of the IL-36 receptor antagonist IL-36Ra (median: 5.42 ng/mL vs. 2.68 ng/mL; *p* < 0.0001), as well as IL-37 (median: 58.19 pg/mL vs. 25.02 pg/mL; *p* < 0.0001) and IL-38 (median: 125.49 pg/mL vs. 49.21 pg/mL; *p* < 0.0001), were significantly elevated in the PG group (Figure 2). The serum concentrations of IL-36 family cytokines and their antagonists in both groups are summarized in Table 2.

### 2.2. Correlation Analysis

Spearman’s rank correlation analysis was performed to assess the relationships between the studied interleukins in the total study population, as well as separately in the patient and control groups. In the patient group, correlations were examined between interleukin levels and selected laboratory and clinical parameters.

In the total study cohort (n = 84), a statistically significant positive correlation was observed between IL-36α and IL-36β (R = 0.354, *p* = 0.0009), as well as between IL-36β and IL-36γ (R = 0.243, *p* = 0.0229). A significant negative correlation was observed between IL-36β and IL-36Ra (R = −0.353, *p* = 0.0009). IL-38 demonstrated a statistically significant negative correlation with IL-36γ (R = −0.237, *p* = 0.0295) and a positive correlation with IL-36Ra (R = 0.215, *p* = 0.0497). Correlations that approached statistical significance included the relationships between IL-36α and IL-36γ (R = 0.207, *p* = 0.059), as well as between IL-36α and IL-36Ra (R = −0.210, *p* = 0.055).

In the control group (n = 40), a statistically significant positive correlation was identified between IL-36α and IL-36β (R = 0.437, *p* = 0.0048).

In the patient group (n = 44), IL-36α showed significantly positively correlated with IL-36β (R = 0.349, *p* = 0.0202), as well as between IL-36β and IL-36γ (R = 0.433, *p* = 0.0034). A statistically significant negative correlation was observed between IL-37 and IL-38 (R = −0.365, *p* = 0.0147). Additionally, a trend toward significance was noted for the negative correlation between IL-37 and IL-36Ra (R = −0.267, *p* = 0.0797).

Correlations between interleukin levels and laboratory or clinical parameters in the patient group revealed several statistically significant associations. IL-36α was negatively correlated with serum IgA concentration (R = −0.308, *p* = 0.0422), as well as with the total ulcer surface area (R = −0.438, *p* = 0.0029). IL-36γ showed a significant negative correlation with white blood cell (WBC) count (R = −0.331, *p* = 0.0284). IL-36Ra demonstrated a negative correlation with total serum protein concentration that approached statistical significance (R = −0.298, *p* = 0.0501). Furthermore, IL-37 was positively correlated with alanine aminotransferase (ALAT) activity (R = 0.400, *p* = 0.0072) and negatively correlated with serum IgM levels (R = −0.303, *p* = 0.0455). IL-38 showed a significant positive correlation with blood glucose concentration (R = 0.406, *p* = 0.0064) (Supplementary Table S1).

## 3. Discussion

To the best of our knowledge, this is the first study presenting IL-36α, IL-36β, IL-36γ, IL-36Ra, IL-37, and IL-38 serum levels in patients with PG. Our results showed a significant decrease in IL-36α and IL-36γ serum concentrations in patients compared to controls. Moreover, IL-36 receptor antagonists: IL-36Ra and IL-38 serum levels were significantly higher in PG patients than in controls. IL-37 was also increased in the PG group compared to the HC. Our results are in opposition to findings in other inflammatory dermatoses.

**Figure 3:**
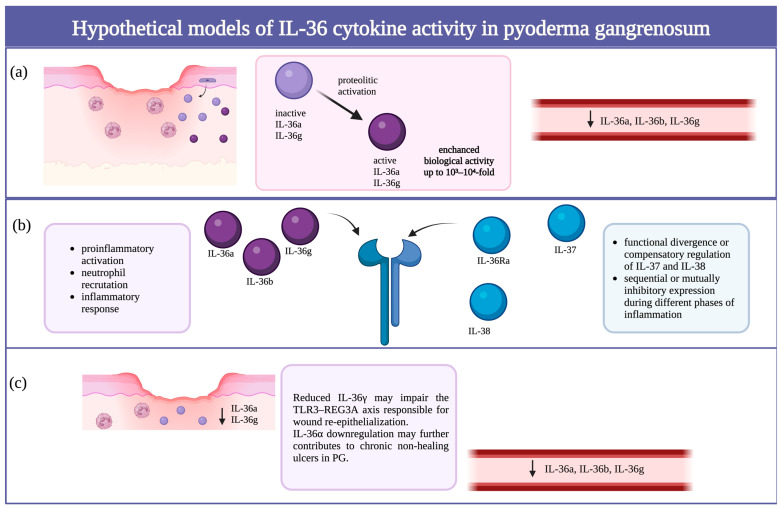
Hypothetical model illustrating the potential roles of IL-36 cytokines in the pathogenesis and impaired wound healing of pyoderma gangrenosum (PG). (**a**) Despite the inflammatory nature of PG, decreased serum levels of IL-36α and IL-36γ may not necessarily reflect reduced cytokine bioactivity. Members of the IL-1 family, including IL-36α, IL-36β, and IL-36γ, are synthesized as inactive precursors that require proteolytic activation to achieve full biological potency—up to 10^3^–10^4^-fold higher than their pro-forms. Thus, local proteolytic processing may enable high IL-36 activity within lesions despite low circulating levels. (**b**) Elevated serum concentrations of IL-36 receptor antagonists (IL-36Ra, IL-37, and IL-38) suggest a compensatory anti-inflammatory response. Negative correlations between IL-37 and IL-38 (*p* = 0.0147) and between IL-37 and IL-36Ra (*p* = 0.0797; trend) indicate potential functional divergence or sequential expression of these cytokines during different phases of inflammation. Their actions may not be synergistic but rather temporally regulated or even mutually inhibitory. (**c**) Reduced IL-36γ may impair the TLR3–REG3A axis responsible for wound re-epithelialization, while lower IL-36α levels negatively correlate with wound size, suggesting a contribution to delayed healing in PG. This unique context of chronic ulceration may account for the distinct IL-36 cytokine profile observed in PG compared with other inflammatory dermatoses lacking ulceration. Created in https://BioRender.com.

The observed decrease in serum levels of IL-36α and IL-36γ in patients with PG, despite the inflammatory nature of the disease, may not necessarily reflect reduced biological activity of these cytokines. It is essential to recognize that members of the IL-1 family, including IL-36α, IL-36β, and IL-36γ, are initially synthesized as biologically inactive precursors that require N-terminal proteolytic cleavage to become fully active. Studies have shown that such truncation can enhance the biological activity of IL-36 cytokines by up to 10^3^–10^4^-fold [17]. In light of this, we hypothesize that despite their relatively low serum concentrations, IL-36α and IL-36γ may exhibit high biological activity at the tissue level, provided they undergo sufficient proteolytic activation. However, this interpretation remains hypothetical, as our study did not assess cytokine levels directly in lesional tissue. Therefore, the proposed model of increased local activity despite lower circulating levels requires validation in tissue-based analyses. This would be consistent with a model in which IL-36-mediated signaling is regulated primarily by post-translational mechanisms and local receptor availability, rather than systemic abundance alone [18]. Such a mechanism could also help explain the paradox of reduced circulating IL-36α/γ in the context of clinically evident inflammation in PG. Since the commercial ELISA kits used in our study likely detect total cytokine concentrations without distinguishing between full-length and truncated forms, the assay may not accurately reflect cytokine bioactivity. It is therefore plausible that the active forms of IL-36α and IL-36γ are present at the site of inflammation (i.e., within skin lesions) but are either rapidly consumed, bound to receptors, or locally degraded, resulting in lower detectable levels in systemic circulation. IL-36 is expressed in the skin by keratinocytes, fibroblasts, endothelial cells, macrophages, DCs, Langerhans cells, and T-cells [19]. Moreover, skin injury increases the expression of IL-36 cytokines in epidermal keratinocytes [20]. Previous observations indicate the involvement of IL-36 in the pathogenesis of PG. Although studies on this topic are still lacking, there has been a report on IL-36 expression in PG lesional skin. A study by Kolios et al. revealed increased expression of genes encoding IL-36α in lesional skin biopsies of PG patients compared to controls [21]. Authors also investigated expression of IL-36γ in lesional skin and controls; however, no difference between groups was observed (Figure 3a).

The significantly elevated serum levels of IL-36Ra, IL-37, and IL-38—natural antagonists of IL-36—suggest a potential compensatory regulatory response aimed at suppressing excessive proinflammatory signaling. Interestingly, we observed negative correlations between IL-37 and both IL-38 (*p* = 0.0147) and IL-36Ra (*p* = 0.0797; trend). Although these cytokines are all considered anti-inflammatory or receptor antagonists within the IL-1 family, the inverse relationship suggests potential functional divergence or compensatory regulation in the inflammatory milieu of PG. One possible explanation is that IL-37 and IL-38 are differentially expressed at distinct phases or intensities of inflammation, and their actions may not be synergistic but rather sequential or even mutually inhibitory. Their differential expression was reported in arthritic diseases and non-small cell lung cancer, indicating functional divergence or compensatory regulation, hinting at non-synergistic, and possibly even sequential or antagonistic actions [22,23] (Figure 3b). It is also conceivable that the observed cytokine profiles might serve as potential biomarkers for monitoring treatment response in PG, although this requires confirmation in prospective studies.

Moreover, recent studies have challenged the exclusively anti-inflammatory role of IL-37. Sullivan et al. demonstrated that proteolytic cleavage of IL-37 by neutrophil-derived proteases can generate a truncated, proinflammatory form that binds the IL-36R, thereby mimicking IL-36 agonists and amplifying inflammation via IL-36R-dependent pathways [24]. This context-dependent dual functionality of IL-37 may be relevant in PG, as disease is characterized by prominent neutrophilic infiltration and protease activity. Thus, although elevated IL-37 levels in serum may reflect a compensatory response, the actual biological effect of IL-37 in PG could be shaped by local proteolytic processing, potentially shifting its role from anti- to proinflammatory depending on the tissue microenvironment.

On the other hand, it should be noted that in PG, in addition to the existing inflammatory state, patients also experience chronic, slow-healing, and prone to recurrence ulcer. Jiang et al. found that the activation of TLR3 leads to the production of IL-36γ, which stimulates keratinocytes to facilitate wound healing by promoting the expression of REG3A or RegIIIg [20]. In our study, we observed decreased levels of IL-36γ, which may indicate the reason for prolonged healing. The role of IL-36α and wound healing has not been reported. In our research, we observed a negative correlation between the wound size and IL-36α concentration. This may indicate that those deviations could potentially be involved in prolonged healing in PG. This unique context of chronic ulceration might also explain why the IL-36 profile in PG differs from that observed in other inflammatory diseases, which typically progress without the presence of wounds (Figure 3c).

There have been case reports describing the successful treatment of PG with spesolimab [25,26]. Spesolimab is a monoclonal antibody that inhibits the IL-36R, thereby preventing downstream proinflammatory signaling, irrespective of circulating IL-36 cytokine levels [27]. Those elucidated IL-36 and related inflammatory storm could decrease through inhibiting IL-36R. Currently, a clinical trial is underway to assess the efficacy of spesolimab in PG management, with an estimated enrollment of approximately 20 participants and an expected completion date of September 2025 [28].

There may be other factors influencing our observations. For instance, a study by Hoffmann et al. found that IL-36Ra autoantibodies decrease IL-36Ra levels in serum/plasma, promote the formation of immune complexes, and enhance unregulated IL-36 signaling [29].

These findings highlight the importance of distinguishing between cytokine quantity and functional activity, and suggest that post-translational modification and local tissue dynamics likely play a crucial role in shaping systemic cytokine profiles in PG. The lack of parallel assessment of IL-36 family cytokines in PG lesional skin is a limitation of the present study. Future studies should evaluate the active forms of IL-36, IL-37, and IL-38 in both serum and in lesional tissue, as this may provide a more comprehensive understanding of their role in PG pathogenesis.

As PG has been described as a component of several autoinflammatory syndromes, including PAPA, PAPASH, PASH, PsAPASH, PASS, and PAC, we carefully reviewed reports on the role of the IL-36 pathway in related dermatological conditions.

In hidradenitis suppurativa (HS), elevated IL-36α, IL-36β, and IL-36γ serum concentrations were reported in a study by Hayran et al. [30]. Increased IL-36 serum levels in HS patients were associated with smoking, obesity, and metabolic syndrome. There is also evidence on the higher expression of IL-36 receptor agonists in the lesional skin of HS patients [31,32,33]. Moreover, increased levels of IL-36Ra have been observed in the lesional skin of patients with HS, whereas IL-37 and IL-38 were found to be elevated in perilesional skin. In contrast, no difference in the lesional expression of IL-36Ra was reported by [31]. Regarding IL-37 and IL-38, the lesional skin exhibited reduced levels of the aforementioned interleukins [33].

In acne, no data regarding serum levels of studied interleukins have been reported to date. However, there have been reports showing increased levels of receptor agonists in lesional skin compared to controls. Gene expression of IL-36Ra in lesional skin was similar to that of the control group [32]. Elevated expression of IL-36, along with reduced IL-38 gene expression in acne patients’ skin samples, was reported. Moreover, they were strongly correlated with increased disease severity [34].

These data collectively suggest that the IL-36 cytokine axis may be dysregulated in a disease-specific manner, with variable patterns of agonist and antagonist expression between autoinflammatory skin conditions [16]. In particular diseases, the secretion of oppositely acting interleukins may be interlinked. In other conditions, a predominant overexpression of proinflammatory mediators is observed.

While none of the included patients had clinically manifest comorbidities at the time of sampling, we cannot entirely exclude the potential impact of subclinical or undiagnosed conditions on interleukin expression profiles.

## 4. Materials and Methods

This case–control study analyzed 44 PG patients recruited in University Centre of General Dermatology and Oncodermatology of Wroclaw Medical University, Poland. As our center is experienced in the diagnosis and treatment of PG and has the status of a tertiary dermatology department in south-west Poland, patients were referred from outpatient clinics for diagnosis verification of ulcerative lesions. Two independent dermatologists were responsible for the evaluation of diagnosis in all participating patients. Patients who achieved a score of 10 or higher on the PARACELSUS score [35] were included in the study. Included patients were diagnosed with ulcerative PG and had inflammatory lesions. Any active comorbidities, malignancies, recent systemic treatment, as well as present infection were among the exclusion criteria as these factors may interfere with the studied parameters. As patients presented to the hospital, the number of skin lesions was counted. All skin lesions were measured, and the area of skin lesions was calculated.

At admission, serum samples were collected from peripheral veins. The concentration of IL-36α, IL-36β, IL-36γ, IL-37, IL-38 and IL-36Ra was determined using the enzyme-linked immunosorbent assay (ELISA) method with commercially available ELISA kits from Biorbyt^®^ (Biorbyt LLC^®^, Durham, NC, USA): Human IL-36A ELISA Kit (Cat. No.: Orb563448), Human IL-36B ELISA Kit (Cat. No.: Orb563450), Human IL-36G ELISA Kit (Cat. No.: Orb562065), Human IL-37 ELISA Kit (Cat. No.: Orb562500), Human IL-1F10/IL-38 ELISA Kit (Cat. No.: Orb562903), and Human IL1RL2/IL-36 Receptor ELISA Kit (Cat. No.: Orb1213095).

The assessment of the receptor and cytokines was performed according to the manufacturer’s protocol and in compliance with good laboratory practice (GLP) standards. Cytokine and receptor concentrations in serum samples were determined by measuring their optical density using an EPOCH microplate spectrophotometer (BioTek^®^ Instruments, Inc., Winooski, VT, USA). The measurement was conducted at a wavelength of 450 nm, with a reference wavelength of 540 nm. The concentration values for individual samples were determined based on standard curves using linear regression, 4-PL nonlinear regression, or automatically generated regression models with Gen5^®^ software.

40 healthy controls (HC), with no history of PG and any other autoimmune or inflammatory diseases, were enrolled as the control group.

### Statistical Analysis

The Shapiro–Wilk test was used to check the data distribution. All the quantitative variables were described as the median and interquartile range (IQR) or mean ± standard deviations (SD). Comparisons between the groups were performed by the Mann–Whitney U test. Correlations between the variables were calculated using Spearman’s rank correlation. A *p*-value less than 0.05 was considered statistically significant. The statistical analysis was performed using Statistica 13.3 software (StatSoft Inc., Tulsa, OK, USA).

The study was conducted in compliance with ethical regulations and follows the principles of the Declaration of Helsinki. The study has been approved by the Bioethics Committee of the Wroclaw Medical University, decision no. KB 235/2023.

## 5. Conclusions

This study provides novel insights into the systemic immunological profile of patients with PG, focusing on both agonists and natural antagonists of the IL-36 cytokine family. It reveals a paradoxical serum cytokine profile, with decreased proinflammatory IL-36α and IL-36γ and increased anti-inflammatory IL-36Ra, IL-37, and IL-38. These changes may reflect compensatory or dysregulated immune responses and could contribute to delayed wound healing. The findings highlight the need to consider both cytokine concentrations and functional status when interpreting systemic inflammatory markers in PG.

Future studies should aim to validate this hypothesis by assessing the active forms of IL-36, IL-37, and IL-38 both in serum and, crucially, within lesional tissue, to better characterize their local biological activity in pyoderma gangrenosum. Given the therapeutic potential of IL-36 pathway blockade, a more detailed understanding of this cytokine network could support the development of targeted treatments for PG and related autoinflammatory syndromes.

## Figures and Tables

**Table 1:** Basic and clinical characteristics of PG patients and healthy controls. (baseline values are presented as mean ± SD or median (IQR) or n%).

	PG Patients	Healthy Controls	*p*-Value
Number of subjects	44	40	-
*Sex*			
Male/female	15 (34.1%)/29 (65.9%)	19(47.5%)/21 (52.5%)	ns
Age (years)(Mean ± SD)	50.2 ± 16.4	48.2 ± 13.0	ns
Area of skin lesions (cm^2^)	19.2 (64.9)	-	-
*Localization of skin lesions*		-	-
Face and neck	1 (2.3%)	-	-
Upper extremity	4 (9.1%)	-	-
Trunk	8 (18.2%)	-	-
Lower extremity	37 (84.1%)	-	-
*Number of lesions*		-	-
1	19 (43.2%)	-	-
2	14 (31.8%)	-	-
3 or more	11 (25.0%)	-	-
*Comorbidities*		-	-
Inflammatory bowel diseases	6 (13.6%)	-	-
Rheumatoid arthritis/spondyloarthropaties	4 (9.1%)	-	-
Systemic lupus erythematous/systemic sclerosis/Sjögren syndrome	2 (4.5%)	-	-

ns—not significant.

**Table 2:** Interleukin (IL)-36α, IL-36β, IL-36γ, IL-36Ra, IL-37, IL-38 serum levels in patients with pyoderma gangrenosum (PG) and control group. Statistically significant associations are shown in bold.

Variables	PG Patients—Median (IQR)	Healthy Controls—Median (IQR)	*p*-Value
IL-36α serum level (ng/mL)	0.9270 (0.9710)	1.6630 (2.6005)	***p* = 0.0003**
IL-36β serum level (pg/mL)	124.4145 (343.3920)	110.4030 (156.6060)	*p* = 0.58
IL-36γ serum level (pg/mL)	23.7660 (45.9335)	52.0420 (70.8810)	***p* = 0.02**
IL-36Ra serum level (ng/mL)	5.4175 (5.3250)	2.6840 (1.5175)	***p* = 0.000**
IL-37 serum level (pg/mL)	58.1910 (40.1045)	25.0155 (25.0235)	***p* = 0.000**
IL-38 serum level (pg/mL)	125.4980 (81.3005)	49.2145 (68.2155)	***p* = 0.000**

PG—pyoderma gangrenosum; IQR—interquartile range.

## Data Availability

The data presented in this study are available on request for scientific purposes from the corresponding author.

## Supplementary Materials

The following supporting information can be downloaded at: https://www.mdpi.com/article/10.3390/ijms262412076/s1.

## Abbreviations

The following abbreviations are used in this manuscript:

IL	Interleukin
PG	Pyoderma gangrenosum
IQR	Interquartile range
HC	Healthy controls
PAPA	PG, acne, and pyogenic arthritis
PAPASH	PG, acne, pyogenic arthritis, and hidradenitis suppurativa
PASH	PG, acne, and hidradenitis suppurativa
PsAPASH	psoriatic arthritis, PG, acne, and hidradenitis suppurativa
PASS	PG, acne, and spondyloarthritis
PAC	PG, acne, and ulcerative colitis
HS	Hidradenitis suppurativa
