# Supplementary material for: Serum Dysregulation of IL-36, IL-37, and IL-38 in Pyoderma Gangrenosum: Clinical Correlations and Implications for IL-36R-Targeted Therapy
Source: Int J Mol Sci. 2025 Dec 15;26(24):12076. doi: 10.3390/ijms262412076 (PMC12732507; doi:10.3390/ijms262412076)
Supplement: Supplementary file 1 [file ijms-26-12076-s001.zip › ijms-3996332-supplementary.pdf]

Suppl. Table 1. Interleukin (IL)-36α, IL-36β, IL-36γ,IL-36Ra, IL-37, IL-38 serum levels and clinical parameters correlations in patients with pyoderma gangrenosum (PG) assesed by Sperman correlation.

Variables	number	R (Spearman)	p
total ulcer surface area & total ulcer surface area			
total ulcer surface area & CRP mg/l	44	-0,016214	0,916801
total ulcer surface area & RBC10*6 /uL	44	0,264533	0,082695
total ulcer surface area & creatinine mg/dL	44	0,248659	0,103607
total ulcer surface area & WBC 10*3 /uL	44	-0,005992	0,969208
total ulcer surface area & HGB g/dL	44	-0,164714	0,285315
total ulcer surface area & glucose mg/dL	44	-0,053582	0,729766
total ulcer surface area & ALAT U/L	44	-0,187725	0,222364
total ulcer surface area & ASPAT U/L	44	-0,220367	0,150603
total ulcer surface area & IgG g/L	44	0,142651	0,355625
total ulcer surface area & IgM g/L	44	0,133380	0,388060
total ulcer surface area & IgA g/L	44	0,296658	0,050533
total ulcer surface area & total serum protein g/dL	44	0,042613	0,783583
total ulcer surface area & IL-36a	44	-0,438148	0,002933
total ulcer surface area & IL-36b	44	0,018749	0,903852
total ulcer surface area & IL-36g	44	0,241128	0,114846
total ulcer surface area & IL-36R	44	0,027489	0,859410
total ulcer surface area & IL-37	44	-0,216247	0,158578
total ulcer surface area & IL-38	44	0,135612	0,380096
total ulcer surface area & number of ulcers	44	0,104820	0,498312
CRP mg/l & total ulcer surface area	44	-0,016214	0,916801
CRP mg/l & CRP mg/l			
CRP mg/l & RBC10*6 /uL	44	-0,342863	0,022704
CRP mg/l & creatinine mg/dL	44	-0,262983	0,084578
CRP mg/l & WBC 10*3 /uL	44	0,292542	0,053976
CRP mg/l & HGB g/dL	44	-0,441391	0,002706
CRP mg/l & glucose mg/dL	44	0,106033	0,493325
CRP mg/l & ALAT U/L	44	0,107176	0,488651
CRP mg/l & ASPAT U/L	44	0,228311	0,136062
CRP mg/l & IgG g/L	44	0,267673	0,078980
CRP mg/l & IgM g/L	44	-0,153613	0,319472
CRP mg/l & IgA g/L	44	0,195798	0,202750
CRP mg/l & total serum protein g/dL	44	0,309071	0,041210
CRP mg/l & IL-36a	44	-0,025657	0,868693
CRP mg/l & IL-36b	44	0,162890	0,290758
CRP mg/l & IL-36g	44	-0,179877	0,242655
CRP mg/l & IL-36R	44	-0,153656	0,319333
CRP mg/l & IL-37	44	0,074925	0,628833
CRP mg/l & IL-38	44	-0,045392	0,769843

CRP mg/l & number of ulcers	44	0,089797	0,562135
RBC10*6 /uL & total ulcer surface area	44	0,264533	0,082695
RBC10*6 /uL & CRP mg/l	44	-0,342863	0,022704
RBC10*6 /uL & RBC10*6 /uL			
RBC10*6 /uL & creatinine mg/dL	44	0,302636	0,045851
RBC10*6 /uL & WBC 10*3 /uL	44	-0,278120	0,067552
RBC10*6 /uL & HGB g/dL	44	0,498006	0,000582
RBC10*6 /uL & glucose mg/dL	44	-0,141121	0,360859
RBC10*6 /uL & ALAT U/L	44	-0,045241	0,770588
RBC10*6 /uL & ASPAT U/L	44	-0,218624	0,153939
RBC10*6 /uL & IgG g/L	44	0,157934	0,305883
RBC10*6 /uL & IgM g/L	44	-0,096979	0,531154
RBC10*6 /uL & IgA g/L	44	0,086556	0,576386
RBC10*6 /uL & total serum protein g/dL	44	-0,075871	0,624494
RBC10*6 /uL & IL-36a	44	-0,174298	0,257818
RBC10*6 /uL & IL-36b	44	0,027985	0,856898
RBC10*6 /uL & IL-36g	44	0,179755	0,242980
RBC10*6 /uL & IL-36R	44	-0,086000	0,578848
RBC10*6 /uL & IL-37	44	-0,085718	0,580098
RBC10*6 /uL & IL-38	44	0,144650	0,348854
RBC10*6 /uL & number of ulcers	44	0,119855	0,438376
creatinine mg/dL & total ulcer surface area	44	0,248659	0,103607
creatinine mg/dL & CRP mg/l	44	-0,262983	0,084578
creatinine mg/dL & RBC10*6 /uL	44	0,302636	0,045851
creatinine mg/dL & creatinine mg/dL			
creatinine mg/dL & WBC 10*3 /uL	44	0,011642	0,940213
creatinine mg/dL & HGB g/dL	44	0,029279	0,850357
creatinine mg/dL & glucose mg/dL	44	-0,234074	0,126189
creatinine mg/dL & ALAT U/L	44	0,012265	0,937019
creatinine mg/dL & ASPAT U/L	44	-0,149532	0,332651
creatinine mg/dL & IgG g/L	44	0,084201	0,586845
creatinine mg/dL & IgM g/L	44	-0,040608	0,793539
creatinine mg/dL & IgA g/L	44	0,176253	0,252434
creatinine mg/dL & total serum protein g/dL	44	0,072785	0,638693
creatinie mg/dL & IL-36a	44	-0,132849	0,389970
creatinine mg/dL & IL-36b	44	0,109775	0,478108
creatinine mg/dL & IL-36g	44	0,207415	0,176705
creatinine mg/dL & IL-36R	44	0,028996	0,851788
creatinine mg/dL & IL-37	44	0,052489	0,735076
creatinine mg/dL & IL-38	44	-0,064200	0,678858
creatinine mg/dL & number of ulcers	44	0,023323	0,880549
WBC 10*3 /uL & total ulcer surface area	44	-0,005992	0,969208
WBC 10*3 /uL & CRP mg/l	44	0,292542	0,053976
WBC 10*3 /uL & RBC10*6 /uL	44	-0,278120	0,067552

WBC 10*3 /uL & creatinine mg/dL	44	0,011642	0,940213
WBC 10*3 /uL & WBC 10*3 /uL			
WBC 10*3 /uL & HGB g/dL	44	-0,110754	0,474165
WBC 10*3 /uL & glucose a mg/dL	44	-0,015681	0,919527
WBC 10*3 /uL & ALAT U/L	44	0,061582	0,691289
WBC 10*3 /uL & ASPAT U/L	44	0,134131	0,385369
WBC 10*3 /uL & IgG g/L	44	-0,277525	0,068166
WBC 10*3 /uL & IgM g/L	44	0,155188	0,314474
WBC 10*3 /uL & IgA g/L	44	0,004195	0,978440
WBC 10*3 /uL & total serum protein g/dL	44	0,004734	0,975667
WBC 10*3 /uL & IL-36a	44	-0,063716	0,681150
WBC 10*3 /uL & IL-36b	44	-0,023611	0,879086
WBC 10*3 /uL & IL-36g	44	-0,330620	0,028379
WBC 10*3 /uL & IL-36R	44	-0,117560	0,447257
WBC 10*3 /uL & IL-37	44	0,068013	0,660903
WBC 10*3 /uL & IL-38	44	-0,003242	0,983336
WBC 10*3 /uL & number of ulcers	44	0,113128	0,464683
HGB g/dL & total ulcer surface area	44	-0,164714	0,285315
HGB g/dL & CRP mg/l	44	-0,441391	0,002706
HGB g/dL & RBC10*6 /uL	44	0,498006	0,000582
HGB g/dL & creatinine mg/dL	44	0,029279	0,850357
HGB g/dL & WBC 10*3 /uL	44	-0,110754	0,474165
HGB g/dL & HGB g/dL			
HGB g/dL & glucose mg/dL	44	-0,154869	0,315483
HGB g/dL & ALAT U/L	44	0,105522	0,495423
HGB g/dL & ASPAT U/L	44	0,030646	0,843456
HGB g/dL & IgG g/L	44	-0,093636	0,545469
HGB g/dL & IgM g/L	44	0,080522	0,603347
HGB g/dL & IgA g/L	44	-0,281091	0,064554
HGB g/dL & total serum protein level g/dL	44	-0,198472	0,196528
HGB g/dL & IL-36a	44	0,078888	0,610743
HGB g/dL & IL-36b	44	-0,125031	0,418707
HGB g/dL & IL-36g	44	-0,117411	0,447838
HGB g/dL & IL-36R	44	-0,000776	0,996010
HGB g/dL & IL-37	44	-0,157418	0,307487
HGB g/dL & IL-38	44	0,046992	0,761961
HGB g/dL &number of ulcers	44	-0,004613	0,976293
glucose mg/dL & total ulcer surface area	44	-0,053582	0,729766
glucose mg/dL & CRP mg/l	44	0,106033	0,493325
glucose mg/dL & RBC10*6 /uL	44	-0,141121	0,360859
glucose mg/dL & creatinine mg/dL	44	-0,234074	0,126189
glucose mg/dL & WBC 10*3 /uL	44	-0,015681	0,919527
glucose mg/dL & HGB g/dL	44	-0,154869	0,315483
glucose mg/dL & glucose mg/dL			

glucose mg/dL & ALAT U/L	44	0,293949	0,052778
glucose mg/dL & ASPAT U/L	44	0,092453	0,550581
glucose mg/dL & IgG g/L	44	0,340541	0,023701
glucose mg/dL & IgM g/L	44	-0,046306	0,765342
glucose mg/dL & IgA g/L	44	0,238096	0,119623
glucose mg/dL & total serum protein g/dL	44	0,376341	0,011808
glucose mg/dL & IL-36a	44	0,045134	0,771119
glucose mg/dL & IL-36b	44	0,108698	0,482460
glucose mg/dL & IL-36g	44	0,023943	0,877396
glucose mg/dL & IL-36R	44	-0,099093	0,522198
glucose mg/dL & IL-37	44	0,405058	0,006382
glucose mg/dL & IL-38	44	-0,137303	0,374129
glucose mg/dL & number of ulcers	44	0,157514	0,307186
ALAT U/L & total ulcer surface area	44	-0,187725	0,222364
ALAT U/L & CRP mg/l	44	0,107176	0,488651
ALAT U/L & RBC10*6 /uL	44	-0,045241	0,770588
ALAT U/L & creatinine mg/dL	44	0,012265	0,937019
ALAT U/L & WBC 10*3 /uL	44	0,061582	0,691289
ALAT U/L & HGB g/dL	44	0,105522	0,495423
ALAT U/L & glucose mg/dL	44	0,293949	0,052778
ALAT U/L & ALAT U/L			
ALAT U/L & ASPAT U/L	44	0,419788	0,004558
ALAT U/L & IgG g/L	44	0,049945	0,747481
ALAT U/L & IgM g/L	44	-0,182323	0,236202
ALAT U/L & IgA g/L	44	0,158932	0,302796
ALAT U/L & total serum protein g/dL	44	0,177033	0,250307
ALAT U/L & IL-36a	44	0,229398	0,134158
ALAT U/L & IL-36b	44	0,052254	0,736216
ALAT U/L & IL-36g	44	0,199979	0,193082
ALAT U/L & IL-36R	44	-0,012075	0,937992
ALAT U/L & IL-37	44	0,399535	0,007213
ALAT U/L & IL-38	44	-0,101473	0,512204
ALAT U/L & number of ulcers	44	0,231530	0,130479
ASPAT U/L & total ulcer surface area	44	-0,220367	0,150603
ASPAT U/L & CRP mg/l	44	0,228311	0,136062
ASPAT U/L & RBC10*6 /uL	44	-0,218624	0,153939
ASPAT U/L & creatinine mg/dL	44	-0,149532	0,332651
ASPAT U/L & WBC 10*3 /uL	44	0,134131	0,385369
ASPAT U/L & HGB g/dL	44	0,030646	0,843456
ASPAT U/L & glucose mg/dL	44	0,092453	0,550581
ASPAT U/L & ALAT U/L	44	0,419788	0,004558
ASPAT U/L & ASPAT U/L			
ASPAT U/L & IgG g/L	44	0,077771	0,615820
ASPAT U/L & IgM g/L	44	-0,058967	0,703787

ASPAT U/L & IgA g/L	44	-0,025109	0,871477
ASPAT U/L & total serum protein g/dL	44	0,195880	0,202556
ASPAT U/L & IL-36a	44	0,216699	0,157687
ASPAT U/L & IL-36b	44	0,113889	0,461668
ASPAT U/L & IL-36g	44	-0,066582	0,667619
ASPAT U/L & IL-36R	44	-0,030643	0,843468
ASPAT U/L & IL-37	44	0,039822	0,797451
ASPAT U/L & IL-38	44	-0,144320	0,349965
ASPAT U/L & number of ulcers	44	0,153224	0,320714
IgG g/L & total ulcer surface area	44	0,142651	0,355625
IgG g/L & CRP mg/l	44	0,267673	0,078980
IgG g/L & RBC10*6 /uL	44	0,157934	0,305883
IgG g/L & creatinine mg/dL	44	0,084201	0,586845
IgG g/L & WBC 10*3 /uL	44	-0,277525	0,068166
IgG g/L & HGB g/dL	44	-0,093636	0,545469
IgG g/L & glucose mg/dL	44	0,340541	0,023701
IgG g/L & ALAT U/L	44	0,049945	0,747481
IgG g/L & ASPAT U/L	44	0,077771	0,615820
IgG g/L & IgG g/L			
IgG g/L & IgM g/L	44	-0,110602	0,474778
IgG g/L & IgA g/L	44	0,180648	0,240607
IgG g/L & total serum protein g/dL	44	0,497209	0,000596
IgG g/L & IL-36a	44	-0,057038	0,713055
IgG g/L & IL-36b	44	0,202341	0,187768
IgG g/L & IL-36g	44	-0,016427	0,915714
IgG g/L & IL-36R	44	0,029470	0,849392
IgG g/L & IL-37	44	0,084673	0,584743
IgG g/L & IL-38	44	0,005499	0,971739
IgG g/L & number of ulcers	44	0,069772	0,652681
IgM g/L & total ulcer surface area	44	0,133380	0,388060
IgM g/L & CRP mg/l	44	-0,153613	0,319472
IgM g/L & RBC10*6 /uL	44	-0,096979	0,531154
IgM g/L & creatinine mg/dL	44	-0,040608	0,793539
IgM g/L & WBC 10*3 /uL	44	0,155188	0,314474
IgM g/L & HGB g/dL	44	0,080522	0,603347
IgM g/L & glucose mg/dL	44	-0,046306	0,765342
IgM g/L & ALAT U/L	44	-0,182323	0,236202
IgM g/L & ASPAT U/L	44	-0,058967	0,703787
IgM g/L & IgG g/L	44	-0,110602	0,474778
IgM g/L & IgM g/L			
IgM g/L & IgA g/L	44	-0,341007	0,023498
IgM g/L & total serum protein g/dL	44	-0,124978	0,418905
IgM g/L & IL-36a	44	0,000705	0,996377
IgM g/L & IL-36b	44	0,075489	0,626246

IgM g/L & IL-36g	44	0,040317	0,794987
IgM g/L & IL-36R	44	0,056881	0,713812
IgM g/L & IL-37	44	-0,303084	0,045515
IgM g/L & IL-38	44	0,161339	0,295437
IgM g/L & number of ulcers	44	0,321090	0,033571
IgA g/L & total ulcer surface area	44	0,296658	0,050533
IgA g/L & CRP mg/l	44	0,195798	0,202750
IgA g/L & RBC10*6 /uL	44	0,086556	0,576386
IgA g/L & creatinine mg/dL	44	0,176253	0,252434
IgA g/L & WBC 10*3 /uL	44	0,004195	0,978440
IgA g/L & HGB g/dL	44	-0,281091	0,064554
IgA g/L & glucose mg/dL	44	0,238096	0,119623
IgA g/L & ALAT U/L	44	0,158932	0,302796
IgA g/L & ASPAT U/L	44	-0,025109	0,871477
IgA g/L & IgG g/L	44	0,180648	0,240607
IgA g/L & IgM g/L	44	-0,341007	0,023498
IgA g/L & IgA g/L			
IgA g/L & total serum protein g/dL	44	0,312437	0,038941
IgA g/L & IL-36a	44	-0,307719	0,042152
IgA g/L & IL-36b	44	0,010292	0,947133
IgA g/L & IL-36g	44	0,088612	0,567330
IgA g/L & IL-36R	44	0,093194	0,547379
IgA g/L & IL-37	44	0,132812	0,390105
IgA g/L & IL-38	44	-0,151775	0,325367
IgA g/L & number of ulcers	44	0,187713	0,222396
total serum protein g/dL & total ulcer surface area	44	0,042613	0,783583
total serum protein g/dL & CRP mg/l	44	0,309071	0,041210
total serum protein g/dL & RBC10*6 /uL	44	-0,075871	0,624494
total serum protein g/dL & creatinine mg/dL	44	0,072785	0,638693
total serum protein g/dL & WBC 10*3 /uL	44	0,004734	0,975667
total serum protein g/dL & HGB g/dL	44	-0,198472	0,196528
total serum protein g/dL & glucose mg/dL	44	0,376341	0,011808
total serum protein g/dL & ALAT U/L	44	0,177033	0,250307
total serum protein g/dL & ASPAT U/L	44	0,195880	0,202556
total serum protein g/dL & IgG g/L	44	0,497209	0,000596
total serum protein g/dL & IgM g/L	44	-0,124978	0,418905
total serum protein g/dL & IgA g/L	44	0,312437	0,038941
total serum protein g/dL & total serum protein			
total serum protein g/dL & IL-36a	44	-0,063275	0,683239
total serum protein g/dL & IL-36b	44	0,297182	0,050108
total serum protein g/dL & IL-36g	44	-0,106409	0,491785
total serum protein g/dL & IL-36R	44	-0,247298	0,105573
total serum protein g/dL & IL-37	44	0,105137	0,497005
total serum protein g/dL & IL-38	44	-0,107469	0,487457

total serum protein g/dL & number of ulcers	44	0,181009	0,239652
IL-36a & total ulcer surface area	44	-0,438148	0,002933
IL-36a & CRP mg/l	44	-0,025657	0,868693
IL-36a & RBC10*6 /uL	44	-0,174298	0,257818
IL-36a & creatinine mg/dL	44	-0,132849	0,389970
IL-36a & WBC 10*3 /uL	44	-0,063716	0,681150
IL-36a & HGB g/dL	44	0,078888	0,610743
IL-36a & glucose mg/dL	44	0,045134	0,771119
IL-36a & ALAT U/L	44	0,229398	0,134158
IL-36a & ASPAT U/L	44	0,216699	0,157687
IL-36a & IgG g/L	44	-0,057038	0,713055
IL-36a & IgM g/L	44	0,000705	0,996377
IL-36a & IgA g/L	44	-0,307719	0,042152
IL-36a & total serum protein g/dL	44	-0,063275	0,683239
IL-36a & number of ulcers	44	-0,219536	0,152187
IL-36b & total ulcer surface area	44	0,018749	0,903852
IL-36b & CRP mg/l	44	0,162890	0,290758
IL-36b & RBC10*6 /uL	44	0,027985	0,856898
IL-36b & creatinine mg/dL	44	0,109775	0,478108
IL-36b & WBC 10*3 /uL	44	-0,023611	0,879086
IL-36b & HGB g/dL	44	-0,125031	0,418707
IL-36b & glucose mg/dL	44	0,108698	0,482460
IL-36b & ALAT U/L	44	0,052254	0,736216
IL-36b & ASPAT U/L	44	0,113889	0,461668
IL-36b & IgG g/L	44	0,202341	0,187768
IL-36b & IgM g/L	44	0,075489	0,626246
IL-36b & IgA g/L	44	0,010292	0,947133
IL-36b & total serum protein g/dL	44	0,297182	0,050108
IL-36b & number of ulcers	44	-0,075661	0,625459
IL-36g & total ulcer surface area	44	0,241128	0,114846
IL-36g & CRP mg/l	44	-0,179877	0,242655
IL-36g & RBC10*6 /uL	44	0,179755	0,242980
IL-36g & creatinine mg/dL	44	0,207415	0,176705
IL-36g & WBC 10*3 /uL	44	-0,330620	0,028379
IL-36g & HGB g/dL	44	-0,117411	0,447838
IL-36g & glucose mg/dL	44	0,023943	0,877396
IL-36g & ALAT U/L	44	0,199979	0,193082
IL-36g & ASPAT U/L	44	-0,066582	0,667619
IL-36g & IgG g/L	44	-0,016427	0,915714
IL-36g & IgM g/L	44	0,040317	0,794987
IL-36g & IgA g/L	44	0,088612	0,567330
IL-36g & total serum protein g/dL	44	-0,106409	0,491785
IL-36g & number of ulcers	44	-0,154168	0,317706
IL-36R & total ulcer surface area	44	0,027489	0,859410

IL-36R & CRP mg/l	44	-0,153656	0,319333
IL-36R & RBC10*6 /uL	44	-0,086000	0,578848
IL-36R & creatinine mg/dL	44	0,028996	0,851788
IL-36R & WBC 10*3 /uL	44	-0,117560	0,447257
IL-36R & HGB g/dL	44	-0,000776	0,996010
IL-36R & glucose mg/dL	44	-0,099093	0,522198
IL-36R & ALAT U/L	44	-0,012075	0,937992
IL-36R & ASPAT U/L	44	-0,030643	0,843468
IL-36R & IgG g/L	44	0,029470	0,849392
IL-36R & IgM g/L	44	0,056881	0,713812
IL-36R & IgA g/L	44	0,093194	0,547379
IL-36R & total serum protein g/dL	44	-0,247298	0,105573
IL-36R & number of ulcers	44	0,140609	0,362623
IL-37 & total ulcer surface area	44	-0,216247	0,158578
IL-37 & CRP mg/l	44	0,074925	0,628833
IL-37 & RBC10*6 /uL	44	-0,085718	0,580098
IL-37 & creatinine mg/dL	44	0,052489	0,735076
IL-37 & WBC 10*3 /uL	44	0,068013	0,660903
IL-37 & HGB g/dL	44	-0,157418	0,307487
IL-37 & glucose mg/dL	44	0,405058	0,006382
IL-37 & ALAT U/L	44	0,399535	0,007213
IL-37 & ASPAT U/L	44	0,039822	0,797451
IL-37 & IgG g/L	44	0,084673	0,584743
IL-37 & IgM g/L	44	-0,303084	0,045515
IL-37 & IgA g/L	44	0,132812	0,390105
IL-37 & total serum protein g/dL	44	0,105137	0,497005
IL-37 & number of ulcers	44	-0,056783	0,714285
IL-38 & total ulcer surface area	44	0,135612	0,380096
IL-38 & CRP mg/l	44	-0,045392	0,769843
IL-38 & RBC10*6 /uL	44	0,144650	0,348854
IL-38 & creatinine mg/dL	44	-0,064200	0,678858
IL-38 & WBC 10*3 /uL	44	-0,003242	0,983336
IL-38 & HGB g/dL	44	0,046992	0,761961
IL-38 & glucose mg/dL	44	-0,137303	0,374129
IL-38 & ALAT U/L	44	-0,101473	0,512204
IL-38 & ASPAT U/L	44	-0,144320	0,349965
IL-38 & IgG g/L	44	0,005499	0,971739
IL-38 & IgM g/L	44	0,161339	0,295437
IL-38 & IgA g/L	44	-0,151775	0,325367
IL-38 & total serum protein g/dL	44	-0,107469	0,487457
IL-38 & number of ulcers	44	-0,137238	0,374358
number of ulcers & total ulcer surface area	44	0,104820	0,498312
number of ulcers & CRP mg/l	44	0,089797	0,562135
number of ulcers & RBC10*6 /uL	44	0,119855	0,438376

number of ulcers & creatinine mg/dL	44	0,023323	0,880549
number of ulcers & WBC 10*3 /uL	44	0,113128	0,464683
number of ulcers & HGB g/dL	44	-0,004613	0,976293
number of ulcers & glucose mg/dL	44	0,157514	0,307186
number of ulcers & ALAT U/L	44	0,231530	0,130479
number of ulcers & ASPAT U/L	44	0,153224	0,320714
number of ulcers & IgG g/L	44	0,069772	0,652681
number of ulcers & IgM g/L	44	0,321090	0,033571
number of ulcers & IgA g/L	44	0,187713	0,222396
number of ulcers & total serum protein g/dL	44	0,181009	0,239652
number of ulcers & IL-36a	44	-0,219536	0,152187
number of ulcers & IL-36b	44	-0,075661	0,625459
number of ulcers & IL-36g	44	-0,154168	0,317706
number of ulcers & IL-36R	44	0,140609	0,362623
number of ulcers & IL-37	44	-0,056783	0,714285
number of ulcers & IL-38	44	-0,137238	0,374358
number of ulcers & number of ulcers			
